# Induced responses to grazing by an insect herbivore (*Acentria ephemerella*) in an immature macrophyte (*Myriophyllum spicatum*): an isotopic study

**DOI:** 10.1002/ece3.1624

**Published:** 2015-08-13

**Authors:** Karl-Otto Rothhaupt, Felix Fornoff, Elizabeth Yohannes

**Affiliations:** Limnological Institute, University of KonstanzMainaustrasse 252, D-78464, Konstanz, Germany

**Keywords:** *Acentria*, freshwater macrophytes, herbivory, *Myriophyllum*, nitrogen sectoriality, nutrient reallocation, stable isotope

## Abstract

While the mechanisms by which adult terrestrial plants deploy constitutive and induced responses to grazing pressure are well known, the means by which young aquatic plants defend themselves from herbivory are little studied. This study addresses nitrogen transport in the aquatic angiosperm *Myriophyllum spicatum* in response to herbivore exposure. Nitrogen tracers were used to monitor nitrogen uptake and reallocation in young plants in response to grazing by the generalist insect herbivore *Acentria ephemerella*. Total nitrogen content (N%) and patterns of nitrogen uptake and allocation (δ^15^N) were assessed in various plant tissues after 24 and 48 h. Following 24 h exposure to herbivore damage (Experiment 1), nitrogen content of plant apices was significantly elevated. This rapid early reaction may be an adaptation allowing the grazer to be sated as fast as possible, or indicate the accumulation of nitrogenous defense chemicals. After 48 h (Experiment 2), plants' tips showed depletion in nitrogen levels of ca. 60‰ in stem sections vulnerable to grazing. In addition, nitrogen uptake by grazed and grazing-prone upper plant parts was reduced and nutrient allocation into the relatively secure lower parts increased. The results point to three conclusions: (1) exposure to an insect herbivore induces a similar response in immature *M. spicatum* as previously observed in mature terrestrial species, namely a rapid (within 48 h) reduction in the nutritional value (N%) of vulnerable tissues, (2) high grazing intensity (100% of growing tips affected) did not limit the ability of young plants to induce resistance; and (3) young plants exposed to herbivory exhibit different patterns of nutrient allocation in vulnerable and secure tissues. These results provide evidence of induced defense and resource reallocation in immature aquatic macrophytes which is in line with the responses shown for mature aquatic macrophytes and terrestrial plants.

## Introduction

The twin imperatives of defense against herbivory and competitive growth mean that both aquatic and terrestrial plants arrive at an optimal strategy through condition-dependent selection of either induced or constitutive defenses (Ito and Sakai 2009; Rasmann and Agrawal 2009). Thus, plants respond to herbivore attack in variable and complex ways that balance competition and defense.

Constitutive resistance may be used to circumvent the severity of herbivore damage by either decreasing herbivore fitness or increasing plant fitness (Karban and Baldwin 1997; Kempel et al. 2011). Such mechanisms are fairly well documented in freshwater angiosperms (ref as given in Fornoff and Gross 2014, p.174).

Induced mechanisms of defense are employed by a number of grazing-prone angiosperms to limit damage and include increased plant toughness, reduced nitrogen content, and thus lower nutritional values. Such responses have been shown to both increase plant fitness (induced defense) and reduce growth of the herbivore (induced resistance) (e.g., Meldau et al. 2012). Increasing nutrient storage on the other hand, for example, by elevating concentrations of amino acids, enhances the nutritional quality of plant tissues and thereby their attractiveness to herbivores (Mattson 1980; McClure 1980; Slansky and Rodriguez 1987). Thus, sectoriality in plant nutrient storage, whereby nutrient levels are reduced in important tissues subjected to grazing and elevated in less vulnerable parts of the plant, is an adaptive response to herbivory.

Most investigations of herbivory-induced plant defenses have focused on terrestrial angiosperms and illuminate the principal characteristics of defenses, including mechanisms of actions, effects on herbivores, and how herbivores react in turn. In contrast, induced defenses against herbivory in aquatic plants are hardly studied. Aquatic macrophytes differ from terrestrial counterparts in that they usually exist in two spatially distinct nutrient environments, with their roots in bottom sediment or soil and their vegetative parts submerged in the water column. Such heterogeneous abiotic resource distribution ought to permit the plant some flexibility in nutrient uptake and chemical-related defense strategies (Smith and Barko 1990).

Earlier studies indicate grazing pressure to be higher in aquatic than in terrestrial ecosystems (Gliwicz and Hillbricht-Ilkowska 1972; Valiela 1984; Ricklefs 1990; Cry and Face 1991), and the rate of grazing damage on submersed leaves is reported to be higher than on floating leaves (Cronin et al. 1998). This has led to an assumption that defenses in aquatic plants might be uncommon and that losses are instead compensated for by increased primary production (Eppley 1981; McQueen et al. 1986; Sager and Richman 1991). However, the ecological assessment of spatiotemporal defense mechanisms by Karban (2011) suggests that plastic, induced defense should be particularly advantageous (e.g., over permanent constitutive defense) in variable situations where plants can access cues to predict future conditions, where defenses are costly and when defense is not necessarily essential. These circumstances apply convincingly to freshwater angiosperms such as *Myriophyllum spicatum*, which reproduces by fragmentation and inhabits aquatic systems with highly variable and heterogeneous spatiotemporal distributions of abiotic resources and herbivores.

A recent experimental study investigating the responses of *M. spicatum* to the generalist insect herbivore *Acentria ephemerella* sought to address the gap in knowledge pertaining to aquatic plant defenses and yielded the first evidence of both constitutive and induced mechanisms in a freshwater angiosperm (Fornoff and Gross 2014). The study was conducted using mature *M. spicatum* in both controlled and in seminatural field conditions. The results indicated diverse responses to grazing by *A. ephemerella*, including reallocation of N-containing metabolites. Given the invasive nature of *M. spicatum* outside its native range, and its ability to reproduce readily by fragmentation, this study sought similar induced defense mechanisms in immature specimens subject to short-term intensive grazing by *A. ephemerella*, which is frequently employed as a biological control agent.

We conducted experiments using single element stable isotope ^15^N as nutrient tracer in young specimens of *M. spicatum*, which are more susceptible to damage than mature plants. In two similar laboratory-based setups, we examined the effect of grazing by *A. emphemerella* larvae on total plant nitrogen, nitrogen uptake, and nitrogen allocation. In order to estimate induced nitrogen reallocation, we analyzed the total nitrogen content (N%) of various tissues obtained from young plants after exposure to grazing. Using isotopic labeling with ^15^N, we investigated nitrogen uptake during grazing (δ^15^N) and tracked the fate of nutrients within plants, including the delocalization of nitrogen-containing compounds in upper and lower plant sections. We expected immature *M. spicatum* subjected to grazing to show visible signs of damage and to respond by reallocating nitrogen content between grazed and nongrazed tissues.

## Materials and Methods

Immature shoots of *M. spicatum* were obtained from a herbivore-free stand growing in a concrete mesocosm (2 w × 2 l × 1 d meter) at the Limnological Institute, University of Konstanz, Germany. Ninety individual shoots with tips of equivalent leaf density, maturity (no flowers and no side shoots), and length (ca. 8–10 cm) were collected at random and transported to the laboratory.

### Experiment 1

**Figure 1:**
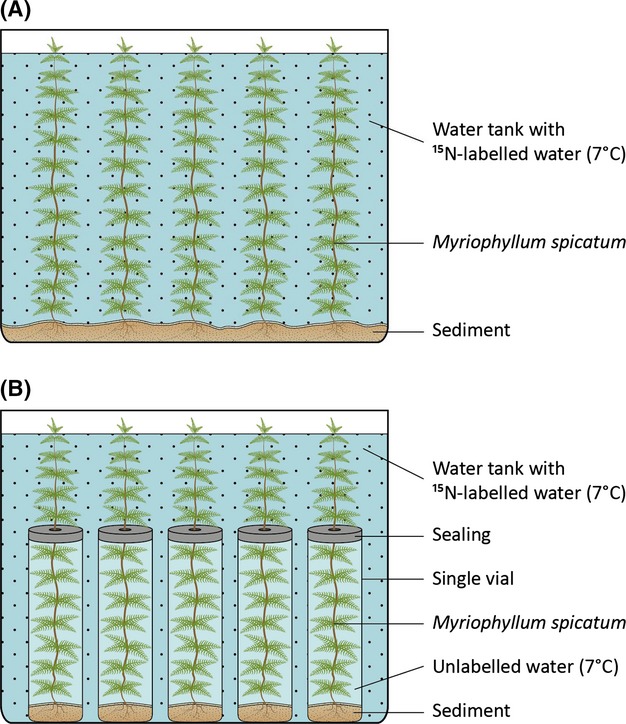
Schematic representation of stable isotope tracer experiment (A) Experiment 1 “Whole tank”) isotope tracer and (B) Experiment 2 “sectorial” isotope tracer experiment.

Thirty *M. spicatum* shoots were planted in a single water tank (38 w × 24 l × 20 d cm) filled with lake water at 7°C (Fig.1A). In order to create rooting substrate for the plants and supply microorganisms and small invertebrates similar to the mesocosm from which the immature shoots of *M. spicatum* were obtained about 50 mL of sieved (5 mm mesh size) sediment was added. The sediment was obtained from the same mesocosm where the plants were collected. To reduce leaching of nutrients from the sediment into the water column, the top of the sediment was covered with a 2 cm layer of clean, fine sand. The tank was then placed in a climate chamber with a 14 h/10 h light and temperature regime of 140/0 μmol quanta·m^−1^·s^−1^ and 24/18°C, respectively.

#### “Whole tank” stable isotope tracer

After allowing plants to take root and grow for 2 weeks, nitrogen tracer (2 mg·L^−1^ ^15^NH_4_Cl Cambridge Isotope Laboratory, 98% atom %) was added to the tank water. Plants had reached a mean height (total length) of ca. 41.39 cm (±5.4, SD). Individual larvae of *A. ephemerella* (instar II, total length of ca. 4–5 mm) were then placed at the apex of every second plant. Samples of plant tissues were collected for stable isotope analysis after 24 h exposure. Plant tips were examined for the presence of clear herbivory signs: feeding scars, feeding larvae, or larval shelters. Based on the presence or absence of all three characteristics, plants were classified as either grazed (G) or nongrazed (NG). Plants with less emphatic signs of damage, including only one or two of these three characteristics were excluded from further analysis. If the herbivore consumed greater portions of the tip or had removed the entire tip, the plant was excluded from the analysis.

Individual plants were gently removed from the sediment and washed with tracer-free lake water. Senescent leaves and roots were removed, and tissue samples were prepared from three locations for stable isotope and nitrogen content analysis. Tip sections (T) comprised the apex including the apical meristem and all nodules (including leafs) separated by internodes shorter than 2 mm (total size of resulting tip was ca. 1 cm³), while the remaining stem was halved into two pieces of equal size yielding upper stem (M) and lower stem (L) sections.

### Experiment 2

Sixty 100-mL crimp-top glass vials were filled with about 20 mL of sieved sediment (5 mm mesh size), covered with ca. 5 mL of fine sand and carefully topped up with lake water. A single *M. spicatum* shoot was planted in each vial, and all vials were placed in a shared water tank (38w × 24 l × 20 d cm) filled with lake water at 7°C. The tank was then maintained in a climate chamber with a 14 h/10 h light and temperature regime of 140/0 μmol quanta·m^−1^·s^−1^ and 24°/18°C, respectively. Plants were incubated for 8 days, after which they had reached a mean height (total length) of ca. 18.5 cm, of which ca. 10.2 cm (±4.4, SD) was inside the vial and ca. 8.3 cm (±3.5, SD) was growth emerging into main body of the tank.

At the end of the incubation period, individual vials were removed gently from the shared tank and the top of the vial was sealed using a soft rubber bung (3 cm diameter × 0.5 cm thickness) with a 3 mm slit and a punched aperture at the center allowing the plant stem to be inserted without damage (Fig.1B). In order to prevent water exchange between the tank and the vial, the plant stem was dried at the point it passed through the ring using soft paper, and the slit and aperture of the rubber bung and the dried plant stem were smeared with silica gel, ensuring a watertight seal between the water of the tank and the vial. The size of the aperture and the softness of the silica gel allowed unimpeded transport of plant fluids and growth of the stem. Following this procedure, plants were able to continue growing with their upper and lower parts isolated in two separate water environments: the lower stems and roots in individual 100 mL crimp-top vials and the upper stems in the shared tank water.

#### “Sectorial” stable isotope tracer

Two liters of tank water was removed, mixed with 1 mg·L^−1^ ^15^NH_4_Cl and returned to the tank, allowing the upper sections of experimental plants to be exposed to the tracer compound, while the lower parts remained isolated in the unlabeled water of the vials. Four days later, a single larva of *A. ephemerella* (instar II, total length of ca. 4–5 mm) was placed at the apical shoot of every second plant. After 48 h, plant-tip (T) and upper stem M) samples were classified as well grazed (G) and nongrazed (NG), as described in Experiment 1, with slightly damaged tissues excluded from analysis.

For sampling of plant tissues, each vial containing an individual plant was removed from the water tank and processed individually. Plant upper sections were cut off at the lowest internode above the rubber seal. The rubber seal was then removed, the plants were cut again at the first internode below the closure, and the lower section of the plant, complete with its roots, was gently removed from the sediment. Each plant part was washed with fresh lake water and kept separately. None of the stem parts tunneling though the rubber showed any signs of damage. The upper parts, which had been exposed to nutrient tracer, were separated into tip samples comprising apices and internodes up to 2 mm in length (T), upper halves of upper stems (S1), and lower halves of upper stems (S2). After removing the root, the lower sections which had not been exposed directly to tracer were cut into two halves ca. 4 cm each. Lower plant samples included the upper halves of lower stems (S3), the lower halves of lower stems (S4), and roots (R).

#### Stable isotope

All samples were dried at 50°C for 48–72 h and pulverized before being subject to stable isotope analysis. Samples were weighed in small tin cups to the nearest 0.001 mg, using a micro-analytical balance and combusted in a vario Micro cube elemental analyzer (Elementar Analysensysteme, Germany). The resulting N_2_ was separated by gas chromatography and passed into a Micromass isotope ratio mass spectrometer (IRMS, Isoprime Ltd., Manchester, UK) for determination of ^15^N/^14^N ratios. Stable isotope values (δ^15^N) are reported in δ notation (per mill) where δ = (1000 × [R_sample_/R_standard_]−1)‰; relative to atmospheric N_2_ for nitrogen in parts per thousand deviations (‰). Two sulfanilamide (Iso-prime internal standards) and two Casein were used as laboratory standards for every 10 unknowns in sequence. Replicate assays of internal laboratory standards indicated measurement errors (SD) of ±0.15‰.

### Statistical analysis

#### Experiment 1 and Experiment 2

For each separate experiment, Two-way analyses of variance were conducted to test for the effects of herbivory (G or NG) and plant section (T, M, L) or (T, S1, S2, S3, S4, R) on nitrogen (N%) or δ^15^N. Bonferroni's multiple test was used for all pairwise comparisons.

## Results

### Nitrogen content (N%) after 24 h – experiment 1

**Figure 2:**
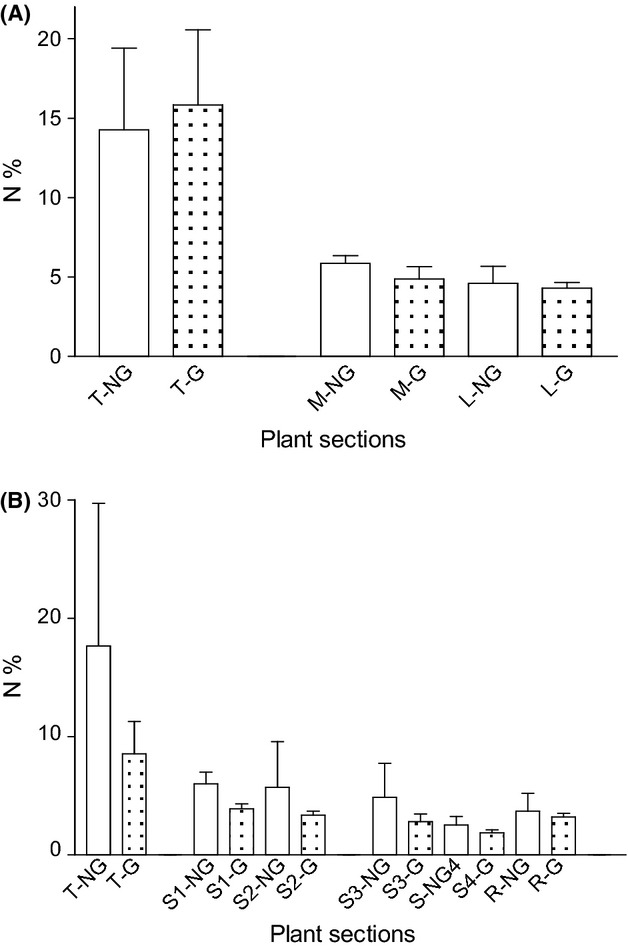
Nitrogen content (N%) of different plant sections of Experiment 1 (A) and 2 (B). Experiment 1: tip (T), upper stem (M), and lower stem (L). Experiment 2: tip (T), upper half of upper stem (S1), lower half of upper stem (S2), upper halve of lower stem (S3), lower half of the lower stem (S4), and root (R).

Results of ANOVA did show a significant effect of plant sections on N% (*F*_2,33_ = 7909; *P* < 0.001) (Fig.2A). Comparisons between three tissues showed significant differences between T and M (*t* = 10.08; *P* < 0.001) and between T and L (*t* = 11.55; *P* < 0.001). Mean N% data indicated that 24 h after herbivore addition, the nitrogen content of grazed tips was slightly elevated (mean N% ± SE: 18.49 ± 1.37) compared to that of nongrazed tips (mean N% ± SE: 14.10 ± 1.08), Fig.2A. Comparisons between M and L plant sections showed no significant difference (*P* = 0.16). However, grazing alone did not have a significant effect on the overall plant N% (*F*_1,33_ = 2.70.22; *P* = 0.12).

### Nitrogen content (N%) after 48 h – experiment 2

After 48 h of exposure to grazing, two-way ANOVA revealed a significant effect of herbivory (*F*_1,156_ = 15.60; *P* < 0.001) on apical N%. However, the effect on tip nitrogen content was reversed compared to that observed at 24 h, with grazed tips showing significantly reduced nitrogen (up to 50%) content (mean N% ± SE: 8.55 ± 0.75) relative to nongrazed tips (mean N% ± SE: 17.66 ± 3.16). The effect of tissue type was also significant (*F*_5,156_ = 15.60; *P* < 0.001). For almost all plant tissues, N% in grazed plants was lower than in nongrazed plants (Fig.2B). Unlike for grazed and nongrazed tip tissues (*t* = 5.30; *P* < 0.001), Bonferroni's pairwise comparisons exhibited no significant difference between grazed and nongrazed S1, S2, S3, S4, and R (*P* > 0.05). In grazed plants, tip tissues (T) exhibited significantly higher N% than S1, S2, S3, S4, and R tissues (*P* < 0.02, for all Bonferroni's pairwise comparisons). All other between-tissue comparisons showed no significance difference (*P* > 0.05). Similarly, in nongrazed plants, N% in T sections was significantly higher than in S1, S2, S3, S4, and R. However, mean (±SE) differences between the nitrogen content (N%) in T and other plant sections were much higher in nongrazed plants (12.1% ± 0.6) than in grazed plants (5.5% ± 0.3). These results, together with those of Experiment 1 (i.e., change in N% in apices within 24 h), confirm that grazing by *A. ephemerella* induces time- and tissue-based variation in nutrient allocation between plant tissues of immature *M. spicatum*. Therefore, in the subsequent nitrogen uptake (δ^15^N) investigations, we questioned whether nitrogen uptake would also be affected by grazing.

### Nitrogen uptake (δ^15^N) over 24 h – experiment 1

#### “Whole tank” stable isotope tracer

Our analyses revealed a significant effect of grazing by *A. ephemerella* on δ^15^N (*F*_2,33_ = 5.81; *P* = 0.025), which varied between plant sections (*F*_2,33_ = 109.26; *P* < 0.001) However, Bonferroni's pairwise comparisons between grazed and nongrazed plant sections showed no significant difference in nitrogen levels after a 24 h of exposure to herbivory (T: *t* = 1.63; *P* = 0.12; M: *t* = 1.36; *P* = 0.19; L: *t* = 1.18; *P* = 0.25). In both grazed and nongrazed plants, a significant reduction in plant δ^15^N values was evident with increased distance from the apex.

**Figure 3:**
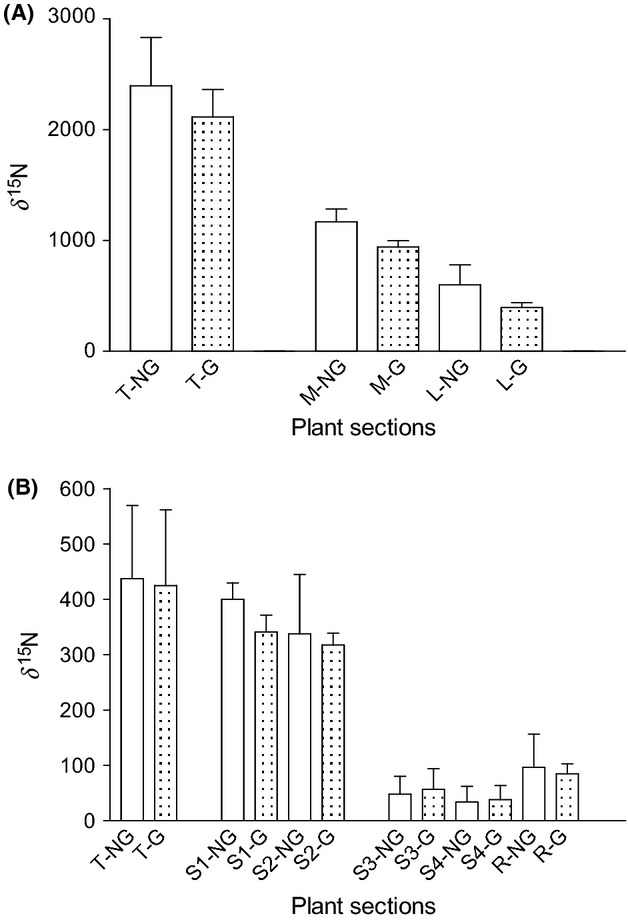
Stable nitrogen isotope (δ^15^N) values of different plant sections of Experiment 1 (A) and 2 (B). Experiment 1: tip (T), upper stem (M), and lower stem (L). Experiment 2: tip (T), upper half of upper stem (S1), lower half of upper stem (S2), upper halve of lower stem (S3), lower half of the lower stem (S4), and root (R).

Immature shoots of *M. spicatum* obtained from the mesocosm stand at the Limnological Institute (University of Konstanz) exhibit a natural δ^15^N of ca. 5‰ (authors unpublished data). Following the addition of nitrogen tracers, all analyzed plant tissues showed a pronounced shift in δ^15^N values, indicating that tracers had been successfully incorporated (in all plant sections, including roots) as a nutrient source for plant growth and competition. Although not statistically significant, 24 h after herbivore addition, δ^15^N of nongrazed tips was elevated compared to grazed tips (Fig.3A). These results indicate that after 24 h of exposure to grazing, nitrogen transport to damaged tips is reduced relative to in nongrazed plants but that nitrogen uptake by damaged tips was not completely suspended.

### Nitrogen uptake (δ^15^N): experiment 2

#### “Sectorial” stable isotope tracer

In Experiment 2, individual plants were incubated in such a way that isotopic tracers imparted “sectorial” δ^15^N labeling only to the upper parts of the plant (T, S1, and S2) (Fig.1B). This allowed us to detect shifts in δ^15^N induced by 48 h of exposure to herbivory. Overall, the isotope values of the exposed upper plant parts were more enriched than those of the isolated lower stems and roots, as shown in Fig.3B.

**Figure 4:**
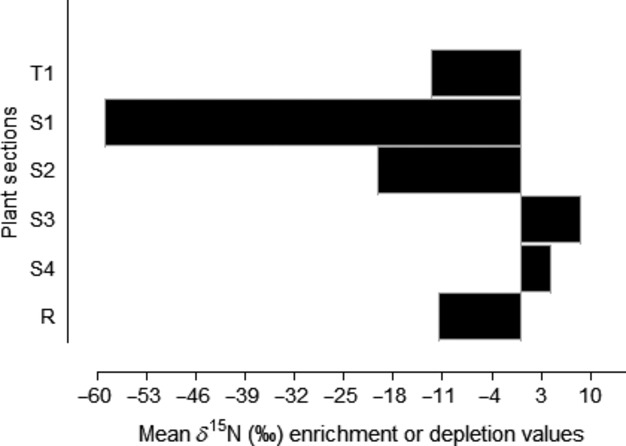
Mean δ^15^N (‰) enrichment or depletion values of different plant sections after 48 h herbivore exposure and tracer addition (Experiment 2).

Following 48 h of exposure, there was no significant effect of herbivory on tissue δ^15^N (*F*_1,156_ = 1.38; *P* = 0.24), but a significant effect of tissue section on δ^15^N was apparent (*F*_5,156_ = 52.58; *P* < 0.001). On average, the δ^15^N values of grazed upper stems sections (S1) were depleted by ca. 60‰ relative to nongrazed S1 stems (Fig.4). This is the part of the plant at closest proximity to the herbivore-damaged tips is therefore likely to be most vulnerable to further grazing. In contrast, the midlower stems (S3) of grazed plants, which are less prone to herbivore damage, showed an enrichment of δ^15^N (mean = ca. 10‰) compared to nongrazed S3 specimens. A minor increase in δ^15^N was also notable in the lowest stem sections (S4) (mean = ca. 4.5‰) of grazed plants. These results indicate an overall reduction in nutrient uptake by grazed and grazing-prone plant parts and a reallocation of nutrient to more secure tissues.

Results of Bonferroni's multiple test for all pairwise comparisons (following two-way ANOVA) on the effects of herbivory (*A. ephemerella*) and plant section nitrogen content (N%) and nitrogen stable isotope (δ^15^N) for Experiments 1 and 2 are given as supplementary materials a and b.

## Discussion

Using inorganic nitrogen isotopes as tracers, we measured plant nitrogen levels (N%) and tracked nitrogen uptake (δ^15^N) by immature submerged specimens of the aquatic macrophyte *M. spicatum* following exposure to an insect herbivore, *A. ephemerella*. Experimentally, grazed plants exhibited three characteristic signs of grazing damage (feeding scars, detectable larvae, and larval shelters), confirming that they had been subject to direct attack. Generally, results showed a negative effect of sustained grazing on the nitrogen content (N%) of most tissues. In agreement with Hempel et al. (2009), our data also indicated reduction in tissue nitrogen levels with increased distance from the growing tips.

### Nitrogen content (N%)

In the first experiment, after 24 h of exposure to *A. ephemerella*, plant tips with characteristic signs of grazing damage exhibited higher nitrogen levels. After 48 h, however, this initial response was reversed and grazed tips exhibited a ca. 50% decline in nitrogen levels compared to nongrazed apices. These time-regulated and tissue- (stem section-) dependent responses could be a mechanism whereby young *M. spicatum* are able to express both induced defense and induced resistance, reducing attractiveness to herbivores while maintaining competitive growth.

Similar experiments using mature *M. spicatum* and longer exposure to *A. ephemerella* have previously resulted in clear induced responses, including changes in the appearance of grazed apices, withdrawal of nitrogen, and increased nitrogen levels in lower parts of grazed shoots (Fornoff and Gross 2014). In mature *M. spicatum*, nitrogen levels in the apices of grazed plants declined by up to 10%, while that of the lower sections was elevated by 14%. The reduction in N% in young plant tips grazed for 48 h in the current study is up to five times greater than observed previously in mature plants (Fornoff and Gross 2014), but the lower sections of immature plants showed no substantial increase in nitrogen levels in either grazed or nongrazed specimens. Despite the lack of elevated nitrogen in secure lower tissues, the stable nitrogen isotope data yielded by the current study (δ^15^N) do imply a faster incorporation of δ^15^N into lower plant sections (see results nitrogen uptake (δ^15^N) – Experiments 1 and 2). It might be expected that large, mature plants with complex structures and multiple growing tips will express a more complex suite of defensive mechanisms incorporating differential uptake, routing, and allocation of nutrients than seen in single-tip immature plants like those in the current investigation. Our isotopic study examined responses to herbivory induced within a maximum of 48 h of grazing exposure. The possibility that longer exposure times might facilitate detection of further induced responses, affecting both uptake and reallocation (storage) of nitrogen-containing compounds in immature plants, remains unexplored.

Previous studies in Lake Constance report that up to 82% of apical meristems in *M. spicatum* specimens with 20–50 tips were damaged or missing, due to grazing by *Acentria* (Gross et al. 2002). Our experimentally grazed plants all had just one tip (simulating the establishment phase of natural propagules), and each individual plant was exposed to just one *Acentria* larva. This could be interpreted as 100% exposure in the grazed treatment, with implications in respect of biocontrol of *M. spicatum* during its establishment and propagation as an alien invader. However, despite this grazing pressure, apices of all experimental plants remained intact, and while stem parts exhibited visible feeding scars, the damage was not severe and there was no fragmentation of apical meristems. Thus, in our experiments, nutrient transport was maintained even during herbivore attack, ensuring the potential for nitrogen-containing metabolites to be allocated to growing meristems and storage organs.

### Nitrogen uptake (δ^15^N)

Our results confirm that exposure to an insect herbivore can result a relatively rapid alteration in nutrient content and nutrient allocation in apical tissues of immature aquatic macrophytes. Within only 24 h of grazer introduction, nongrazed apices were enriched by up to 280‰ compared to grazed shoots (experiment 1, δ^15^N). However, after 48 h of grazing exposure, δ^15^N values were equivalent in grazed and nongrazed apices. The levels of ^15^N enrichment are possibly but not necessarily in the range where simple diffusion and equilibration could result. Nevertheless, the results indicate a reduced rate of nitrogen uptake in grazed plants compared to ungrazed plants and a net transport of nutrient to affected plant parts. This could be interpreted as evidence of concurrent induced defense and induced resistance responses whereby an attacked plant may be able to (1) prevent loss of growing apical meristems, resulting in increased fitness of the plant and (2) limit the nutritional value of the attacked tissues, thereby making the apices less attractive as dietary sources and resulting in decreased fitness of the attacking herbivore.

Presumably, the observed short-term response to onset of grazing reflects an almost immediate uptake or accumulation of nitrogen-rich defensive chemicals such as alkaloids in apical meristems. By acting as a deterrent to the herbivore, such a reaction might be expected to minimize injury and prevent loss of growing apical meristems. Alternatively, a herbivory-induced response that increases N% in apices could also confer defense by sating the herbivore faster, while the potential for rapid compensatory growth by young *M. spicatum* may render localized induced nutrient uptake a cost-effective defense against *A. ephemerella*.

### Herbivore-induced changes in resource allocation and sequestration

Comparisons between six different tissue sections confirmed major responses in nutrient allocation in grazed plants. Nitrogen transport to plant sections closest to the site of herbivore damage was reduced. Secondly, nutrient reallocation to lower sections of the stem was enhanced. Coupling the results of the whole-plant and sectoral stable isotope-labeling experiments, we see that herbivory induces changes to resource allocation and sequestration of nutrient into lower stems. These tissues may serve as secure storage for the limiting resource of nitrogen, including complex nitrogen compounds and nitrogen-rich chlorophyll molecules, while simultaneously making the attacked plants less appealing to herbivores.

The sequestration of nutrient resources in less vulnerable plant parts may bestow both growth and competitive advantages on young plants. The suite of induced responses have previously observed in mature *M. spicatum* (Fornoff and Gross 2014), included nutrient sequestration, and similar strategies are documented in terrestrial plants (e.g., Vergés et al. 2008; Orians et al. 2011; Meldau et al. 2012) and in another submerged macrophyte, *Potamogeton perfoliatus*, following grazing by *A. ephemerlla* larvae (Miler and Straile 2010).

In summary, herbivory places additional demands on plants, through direct appropriation of resources and by altering the plant's functional physiology. Induced withdrawal of nutrients from grazed tissues has implications for the fitness of both the plant and the herbivore. Plants might render themselves less attractive to herbivores by reducing the availability of stored nitrogen and thus the nutritional value of particular tissues. This reduced nutrient intake will impair or halt growth and fecundity in individual herbivores, but it may also encourage higher consumption, which can only be tolerated by fast-growing macrophytes (Orians et al. 2011). A second possibility, when a plant is not resource limited, is for some nutrient to be reallocated from the most important tissues (such as apical meristems) to other tissues, leaving adequate nutrient in targeted tissues for herbivores to feed without causing significant damage or driving it to colonize other sections of the plant. The second argument is more likely to hold if the plant is growing slowly and if grazed or grazing-prone tissue is particularly essential for survival. In our experiment, we used young plants undergoing rapid development, in which nutrient reduction likely to be the most cost-efficient defense mechanism for withstanding damage without a reduction in fitness.

There are increasing evidences on the ecological function of interplant volatile signaling in plant–herbivore interactions (e.g., Heil and Karban 2010; Pearse et al. 2013). Our experimental set-up allows the potential for organic compounds released by the grazed plants to be detected by the ungrazed ones. As the importance of interplant signaling in plant–herbivore interactions has barely been studied in aquatic systems, the direction of its potential effect on the results presented in this study could be a question of future research.

Finally, while patterns of constitutive chemical resistance in aquatic ecosystems have been well studied (e.g., Prusak et al. 2005; Agrawal and Fishbein 2006; Erhard et al. 2007), detailed comparisons between induced resistance mechanisms described in freshwater plants are limited. This is because generalizations about herbivory in aquatic systems have to be based on relatively few data (e.g., Morrison and Hay 2011; Fornoff and Gross^2014^). Thus, far, induced defense has been reported in two recent studies of marine angiosperms (Steele and Valentine 2012; Darnell and Heck 2013) and in a limited number of earlier studies of freshwater angiosperms (Jeffries 1990; Bolser et al. 1998; Lemoine et al. 2009; Morrison and Hay 2011; Fornoff and Gross 2014).

Although all aquatic angiosperms share common ancestors with terrestrial plants and are secondary adapted to water, it is not yet to know whether they exhibit an equivalent capacity for induced and constitutive defense (Cook 1999). This study provides additional isotopic evidence for rapid induced responses to grazing by an insect herbivore (*A. ephemerella*) in an immature macrophyte (*M. spicatum*). This adaptive response could be a key trait supporting the species' successful establishment as an invasive alien in North America. The current work also highlights that accurate interpretation of macrophyte nitrogen content and nitrogen stable isotope values requires a cautious assessment herbivore pressure, as even very short periods of exposure to grazing are sufficient to induce a defensive response.

## Supporting Information

**Appendix S1.** Bonferroni's Multiple Test for all Pairwise comparisons (following Two-way ANOVA) on the effects of herbivore (*A. ephemerella*) and plant section nitrogen content (N%) and nitrogen stable isotope (δ^15^N) for experiment 1 and 2.
