# Supplementary material for: Induced responses to grazing by an insect herbivore (Acentria ephemerella) in an immature macrophyte (Myriophyllum spicatum): an isotopic study
Source: Ecol Evol. 2015 Aug 13;5(17):3657–65. doi: 10.1002/ece3.1624 (PMC4567869; doi:10.1002/ece3.1624)
Supplement: Supplementary file 1 — Appendix S1. Bonferroni's Multiple Test for all Pairwise comparisons (following Two-way ANOVA) on the effects of herbivore (A. ephemerella) and plant section nitrogen content (N%) and nitrogen stable isotope (δ15N) for experiment 1 and 2. [file ece30005-3657-sd1.docx]

**Supplementary Materials**

a) Bonferroni's Multiple Test for all Pairwise comparisons (following Two-way ANOVA) on the effects of herbivore (*A. ephemerella*) and plant section nitrogen content (N%) and nitrogen stable isotope (δ^15^N).

Experiment 1: tip (T); upper stem (M); and lower stem (L).

*P < 0.05, **P < 0.01, ***P < 0.001.

**___________________________________________________________________________**

**Experiment 1 N%**

**Plant Section t P**

T~M 10.08*** <0.001

T~L 11.55*** <0.001

M~L 1.47 0.16

**-------------------------------------------------------------------------------------------------------------------**

**Experiment 1 δ^15^N**

**Herbivory**

**(Non-Grazed vs. Grazed)** **t P**

T 1.63 0.12

M 1.36 0.19

L 1.18 0.25

**--------------------------------------------------------------------------------------------------------------------**

**Experiment 1 δ^15^N**

**Plant Section Non-Grazed Grazed**

**t P t P**

T~M 7.56*** <0.001 6.51*** <0.001

T~L 11.08*** <0.001 9.48*** <0.001

M~L 3.51** <0.01 2.97** 0.01

------------------------------------------------------------------------------------------------------------------

b) Bonferroni's Multiple Test for all Pairwise comparisons (following Two-way ANOVA) on the effects of herbivore (*A. ephemerella*) and plant section nitrogen content (N%) and nitrogen stable isotope (δ^15^N) for experiment 1and 2.

Experiment 2: tip (T1), upper half of upper stem (S1), lower half of upper stem (S2), upper halve of lower stem (S3), lower half of the lower stem (S4) and root (R).

*P < 0.05, **P < 0.01, ***P < 0.001.

**______________________________________________________________**

**Experiment 2 N%**

**Herbivory**

**(Non-Grazed vs. Grazed)** **t P**

T1 5.30*** <0.001

S1 1.27 0.20

S2 1.11 0.27

S3 1.40 0.16

S4 0.33 0.74

R 0.23 0.82

**--------------------------------------------------------------------------------------------------------------**

**Experiment 2 N%**

**Plant Section Non-Grazed Grazed**

**t P t P**

T1~S1 7.15*** <0.001 3.12* 0.02

T1~S2 8.00*** <0.001 3.81** 0.003

T1~S3 7.38*** <0.001 3.45** 0.009

T1~S4 9.45*** <0.001 4.56*** <0.001

T1~R 8.53*** <0.001 3.45** 0.009

S1~S2 0.85. 0.87 0.69 0.99

S1~S3 0.23 0.82 0.36 1.00

S1~S4 0.91 0.89 1.01 0.95

S1~R 1.38 0.73 0.02 0.98

S2~S3 0.63 0.90 0.33 0.93

S2~S4 1.44 0.73 0.67 0.98

S2~R 0.53 0.8 0.35 0.99

S3~S4 2.07 0.31 1.00 0.95

S3~R 1.16 0.82 0.02 0.98 S4~R 0.91 0.89 1.03 0.96

-----------------------------------------------------------------------------------------------

**Experiment 2 δ^15^N**

**Plant Section t P**

T1~S1 1.94 0.20

T1~S2 12.06*** <0.001

T1~S3 3.33 0.01

T1~S4 9.35*** <0.001

T1~R 10.95*** <0.001

S1~S2 10.12*** <0.001

S1~S3 1.39 0.31

S1~S4 7.39*** <0.001

S1~R 9.01*** <0.001

S2~S3 8.73*** <0.001

S2~S4 2.82* 0.03

S2~R 1.11 0.27

S3~S4 5.99*** <0.001

S3~R 7.62*** <0.001

S4~R 1.70 0.25

------------------------------------------------------------------------------------------------
